# Distinct roles of DKK1 and DKK2 in tumor angiogenesis

**DOI:** 10.1007/s10456-013-9390-5

**Published:** 2013-10-04

**Authors:** Hongryeol Park, Hyei Yoon Jung, Hyun-Jung Choi, Dong Young Kim, Ji-Young Yoo, Chae-Ok Yun, Jeong-Ki Min, Young-Myoung Kim, Young-Guen Kwon

**Affiliations:** 1 Department of Biochemistry, College of Life Science and Biotechnology, Yonsei University, Seoul, 120-752 Republic of Korea; 2 Department of Bioengineering, College of Engineering, Hanyang University, Seoul, South Korea; 3 Research Center for Integrative Cellulomics, Korea Research Institute of Bioscience and Biotechnology, 125 Gwahak-ro, Yuseong-gu, Taejon, 305-806 Republic of Korea; 4 Vascular System Research Center, Kangwon National University, Chuncheon, Kangwon-Do 200-701 Republic of Korea

**Keywords:** DKK1, DKK2, Tumor angiogenesis, Perivascular coverage, Vessel normalization

## Abstract

Tumor angiogenesis is essential for tumor invasive growth and metastasis, and generates abnormal vascular structures unlike developmental neovessel formation. To reduce tumor vascular abnormalities such as leakage and perivascular cell coverage deficiency that limit cancer therapy effectiveness, novel therapeutic approaches focus on vessel normalization. We have previously shown that Dickkopf-1 (DKK1), a Wnt antagonist, inhibits and its homolog DKK2 enhances, angiogenesis in normal tissues. In the present study, we investigated the effects of DKK1 and DKK2 on tumor growth and angiogenesis. Treatment of B16F10 melanoma-bearing mice with adenovirus expressing DKK1 significantly reduced tumor growth but DKK2 increased growth compared with controls. Similar pattern of tumor growth was observed in endothelial-specific DKK1 and DKK2 transgenic mice. Interestingly, tumor vascular density and perfusion were significantly decreased by DKK1 but increased by DKK2. Moreover, coverage of blood vessels by pericytes was reduced by DKK1, while DKK2 increased it. We further observed that DKK1 diminished retinal vessel density and increased avascular area in an in vivo murine model of oxygen-induced retinopathy, whereas DKK2 showed opposite results. These findings demonstrate that DKK1 and DKK2 have differential roles in normalization and functionality of tumor blood vessels, in addition to angiogenesis.

**Electronic supplementary material:**

The online version of this article (doi:10.1007/s10456-013-9390-5) contains supplementary material, which is available to authorized users.

## Introduction

Tumor angiogenesis is required for tumor development and supports rapid tumor growth [1]. Because tumor growth is robust, oxygen and nutrient supply by newly-formed vessels are often insufficient for optimal growth [2]. The hypoxic tumor environment induces secretion of growth factors including vascular endothelial growth factor (VEGF). Excessively expressed growth factors disrupt normal angiogenic processes in tumors [3]. Tumor vessels show abnormal vascular features such as leakage, poor vascular coverage, and disorganized sprouting that inhibit vascular perfusion and increase tumor metastasis risk. Although diverse angiogenic molecules are implicated in tumor angiogenesis, to date, VEGF has been the primary target in developing novel anti-angiogenic therapeutic applications. However, because targeting VEGF alone has reported limitations in mitigating tumor growth, the study of other angiogenic factors is markedly growing [4].

Wnts are secreted glycoproteins that regulate diverse cellular and biological processes such as proliferation, survival, migration, and development of various tissues, through both beta-catenin-dependent and -independent mechanisms [5]. Wnt signaling is modulated by diverse receptors and antagonists such as Frizzled, LRP, sFRP, and the Dickkopf (DKK) family members [6]. There are emerging reports that the Wnt signaling pathways and their antagonists modulate vessel neogenesis, including during developmental vasculogenesis and pathological angiogenesis [7]. We previously demonstrated distinct roles of DKK1 and DKK2, known Wnt antagonists, in mediating developmental angiogenesis [8]. DKK1 inhibits human umbilical vein endothelial cell (EC) tube formation and proliferation by reducing both Wnt/beta-catenin and DKK2/cell division-control protein 42 (CDC42) signaling activity. Transgenic mice that selectively overexpress murine DKK1 in ECs (DKK1 Tg) show retarded retinal and bone angiogenesis [8, 9]. Conversely, DKK2, a DKK1-homolog, enhances EC migration by activating CDC42 independent of Wnt. DKK2 improves recovery from hind limb ischemia and cardiac infarction by enhancing angiogenesis [8].

It is known that activity of Wnt and their antagonists, including DKK family members, correlates with patient prognosis with different cancer cell types [6]. In renal carcinoma, DKK1 and DKK2 expression is epigenetically suppressed, and their ectopic expression induces apoptosis, and decreases invasion, of cancer cells [10, 11]. DKK1 inhibits tumor growth by activating apoptosis of MDA-MB435 melanoma cells [12]. DKK2 increases tumor growth and metastasis through transcriptional upregulation of matrix metalloprotease-1 in Ewing’s Sarcoma [13]. Although these results demonstrate that DKK1 and DKK2 regulate growth of some tumor cell types, the effects of DKK1 and DKK2 on tumor angiogenesis has not been thoroughly studied.

Recent anti-neoplastic strategies have focused on normalizing the tumor vasculature integrity and perivascular cell coverage, to improve therapeutic drug delivery [14–18]. Overexpressed platelet-derived growth factor-D (PDGF-D) normalizes tumor vessels by enhancing perivascular coverage, thereby facilitating anti-cancer drug delivery and efficacy [19]. Angiopoientin-1 enhances vessel maturation by enhancing extravascular pericytes coverage and strengthening EC–EC junctions, whereas angiopoietin-2 inhibits this improvement [20]. Growing evidence supports the hypothesis that DKK1 and DKK2 regulate vascular stability. DKK1 inhibits β-catenin accumulation that is a component of endothelial junctional complexes, suggesting that DKK1 might increase vascular leakage [21]. Wnt canonical signaling induces pericyte and smooth muscle cell (SMC) proliferation, and is inhibited by DKK1 [22–24]. On the contrary, DKK2 induces normal morphology vessel sprouting compared to VEGF-induced angiogenesis in a cornea pellet implant assay [8]. However, there currently exist few reports of DKK1 and no direct reports of DKK2 involvement in vessel normalization.

In the present study, we investigated the effects of DKK1 and DKK2 in tumor growth and angiogenesis in a murine model of B16F10 melanoma. Using an adenoviral infection and EC-specific transgenic mice, we show that DKK1 and DKK2 differentially regulate structure and functionality of tumor blood vessels, in addition to tumor angiogenesis.

## Methods

### Generation of mDKK1 and mDKK2 adenoviruses

To generate adenoviruses expressing the mDKK1 or mDKK2 genes, we constructed two kinds of adenoviral E1 shuttle vectors, called pCV14-mDKK1 or PCV14-mDKK2, respectively. Linearized shuttle vectors were co-transformed into *Escherichia coli* BJ5183 along with the linearized adenoviral vector, vmdl324Bst, for homologous recombination, generating pdl-mDKK1 or pdl-mDKK2, respectively [25]. The verified recombinant adenoviral plasmid DNA was digested with *Pac*I and transfected into HEK293T cells to generate the Ad-mDKK1 and Ad-mDKK2 vectors. All viruses were propagated in HEK293T cells and purified by CsCl density purification, dissolved in storage buffer (10 mM Tris–HCl, 4 % sucrose, 2 mM MgCl_2_), and stored at −80 °C. Viral particle numbers were calculated by measuring optical density at 260 nm, where 1 absorbency unit is equivalent to 10^12^ viral particles/ml), and infectious titers (plaque-forming units/ml) were determined by limiting dilution assay on HEK293T cells. The multiplicity of infection was calculated from infectious titers.

### DKK1 and DKK2 overexpression in B16F10 cells

For DKK1 and DKK2 overexpression, cDNAs encoding DKK1 and DKK2 were synthesized and subcloned into a lentiviral vector (Macrogen, Seoul, South Korea). To generate stable transfectants, these vectors were introduced into HEK293T cells that were grown in high-glucose Dulbecco’s Modified Eagle’s medium (DMEM) containing 10 % fetal bovine serum (FBS), with packaging vectors using Lipofectamine™ (Life Technologies Corp., Carlsbad, CA), according to manufacturer instructions. The next day, viruses in cell supernatants were added to B16F10 cells along with 5 μg/ml polybrene^®^ (Sigma Aldrich, St. Louis, MO). After 24 h, media were replaced with fresh media containing 1.2 μg/ml puromycin. Puromycin-resistant clones were selected by incubating cells for 2 weeks in medium containing puromycin.

### Cell proliferation assay

Stable transfectants (5 × 10^4^ cells) were seeded in six-well tissue culture plates in 2 ml DMEM containing 5 % FBS/well. After 3, 6, 9, or 12 days, viable cells were counted in a cell counter (Innovatis GmbH, Dusseldorf, Germany).

### Soft agar colony-forming assay

Anchorage-independent growth assays were performed using the CytoSelect™ 96-well Cell Transformation Assay kit (Cell Biolabs, Inc., San Diego, CA). Stable DKK1 or DKK2 transfectants (5 × 10^4^ cells) were plated in DMEM containing 10 % FBS in a cell-suspension agar matrix between layers of base agar matrix. After 3 weeks, the agar matrix was solubilized, cells were stained with MTT solution, and absorbance at 570 nm was measured using a microplate reader. Experiments were performed in triplicate wells/condition.

### Apoptosis assay

Stable transfectants (5 × 10^4^ cells) were seeded in six-well tissue culture plates in 2 ml DMEM containing 5 % FBS/well. After 6 or 12 days, cells were washed with ice-cold phosphate-buffered saline (PBS) and resuspended in 100 mM HEPES buffer, pH 7.4, containing protease inhibitors (5 mg/ml aprotinin and pepstatin, 10 mg/ml leupeptin, and 0.5 mM phenylmethanesulfonyl fluoride). Cell suspensions were lysed by three freeze–thaw cycles, and the cytosolic fraction was obtained by centrifugation at 12,000×*g* for 20 min at 4 °C. DEVDase activity was evaluated by measuring proteolytic cleavage of chromogenic substrate, Ac-DEVD-pNA, a substrate for caspase-3-like proteases. Cell lysates (80 μg protein) were added to assay buffer containing 150 M Ac-DEVD-pNA to a final volume of 150 μl. The mixture was incubated at 37 °C for 1 h, and enzymatically-released pNA was measured at 405 nm in a microplate reader every 20 min.

### Tumor analysis

B16F10 murine melanoma cells (5 × 10^5^ cells; syngeneic to C57BL/6 mice) in 100 μl Hank’s balanced salt solution were injected subcutaneously into the abdominal area of 6- to 8-week-old male C57BL/6 mice. After tumors appeared in the mice, tumor growth was monitored at 2-day intervals by measuring the length and width of the tumor with a caliper and calculating tumor volume on the basis of the following formula: volume (mm^3^) = 0.523*Lw* ^2^. The percentage of surviving mice was determined by monitoring the tumor growth-related events (tumor size >3,000 mm^3^) over a period of 20 days.

### Adenovirus tumor model

When tumors generated in mice were 60–70 mm^3^ in volume, Ad-ΔE1, Ad-ΔE1-mDKK1, or Ad-ΔE1-mDKK2 mixed with Lipofectamine ™Plus solution (Life Technologies Corp./Gibco BRL, Carlsbad, CA) at a 2:6 ratio were administered intratumorally (5 × 10^8^ plaque-forming units/tumor in 30 μl PBS).

### Cryosectioning and immunofluorescence staining

Tissues were fixed in 4 % paraformaldehyde (PFA)-PBS (pH 7.4) overnight at 4 °C and rinsed with PBS at room temperature. Tissues were incubated in 15 % sucrose overnight at 4 °C and transferred to 30 % sucrose at 4 °C until the tissue sank. Tissues were infiltrated with Tissue Tek^®^ OCT™ embedding medium for 0.5 h at room temperature before freezing. Tissues were transferred to an embedding mold filled with OCT™, and were frozen in liquid nitrogen, followed by storage at −70 °C. Sections (8- to 50-μm thick) were cut at −20 °C, and slides were stored at −70 °C until needed. Sections were prefixed in acetone for 0.5 h at −70 °C and briefly air-dried. OCT was removed with water, and sections were incubated in blocking solution for 4 h at 37 °C or overnight at 4 °C, followed by overnight incubation in primary antibody at 4 °C. After five washes in 0.3 % Triton-X-100 in PBS for 15 min each, sections were incubated in secondary antibody overnight at 4 °C. Sections were treated with DAPI (1 μg/ml) and washed five more times with 0.3 % Triton-X-100 in PBS for 15 min each. All antibodies were dissolved in antibody diluent (Dako, Glostrup, Denmark). Sections were analyzed by fluorescence microscopy using an Olympus IX81-ZDC inverted fluorescence microscope or a confocal microscope (Carl Zeiss, LSM 510 META and LSM 700 META).

### Lectin perfusion assay

Functional tumor vessels were labeled in mice by i.v. injection of 100 μl of biotinylated *Lycopersicon esculentum* (tomato) lectin (1 mg/ml; Vector Labs, Burlingame, CA). After 30 min circulation time, mice were anesthetized with Avertin^®^ (tribromoethanol), and whole-body perfusion fixation was performed with 1 % PFA/PBS. Tumors were excised and prepared for cryosectioning and immunofluorescence staining.

### OIR model

Seven day-old mice were exposed to a hyperoxic atmosphere (75 % O_2_) for 5 days, followed by return to normoxia for an additional 5 days. Mice were anesthetized with Avertin^®^ and eyes were removed and fixed in 4 % PFA-PBS (pH 7.4) for 1 h at 4 °C. Retinas were dissected, postfixed in 1 % PFA for 2 h at room temperature, washed with PBS, and permeabilized with PBS containing 1 % Triton-X-100 for 1 h. Retinas were incubated in blocking solution for 4 h at 37 °C, followed by overnight incubation in Alexa488-conjugated isolectin GS-IB_4_ solution (Molecular Probes/Invitrogen Corp., Eugene, OR) at 4 °C. After five washes in PBS containing 1 % Triton-X-100, retinas were flat-mounted on slides and analyzed using an Olympus IX81-ZDC inverted fluorescence microscope.

### Animal studies

All mice were maintained in a laminar airflow cabinet under specific pathogen-free conditions. All facilities were approved by the Association of Assessment and Accreditation of Laboratory Animal Care and all animal experiments were conducted under the institutional guidelines established for the Animal Core Facility at Yonsei University College of Medicine.

### Vessel density analyses

The vessel density of tumor and retina, and hypoxic region of tumor were determined by using Multi Gauge Fujifilm (Tokyo, Japan).

### Statistical analyses

Data are presented as mean ± standard deviation (SD) or ±standard error (SE), as indicated. Statistical comparisons between groups were performed using one-way analysis of variance followed by Student’s *T* test. Survival curves were plotted against time after treatment (Kaplan–Meier survival function) and compared using a log-rank test analysis (Stat View software; Abacus Concepts Inc., Berkeley, CA).

## Results

### Ad-DKK1 inhibits and Ad-DKK2 enhances tumor growth by modulating tumor angiogenesis

**Fig. 1:**
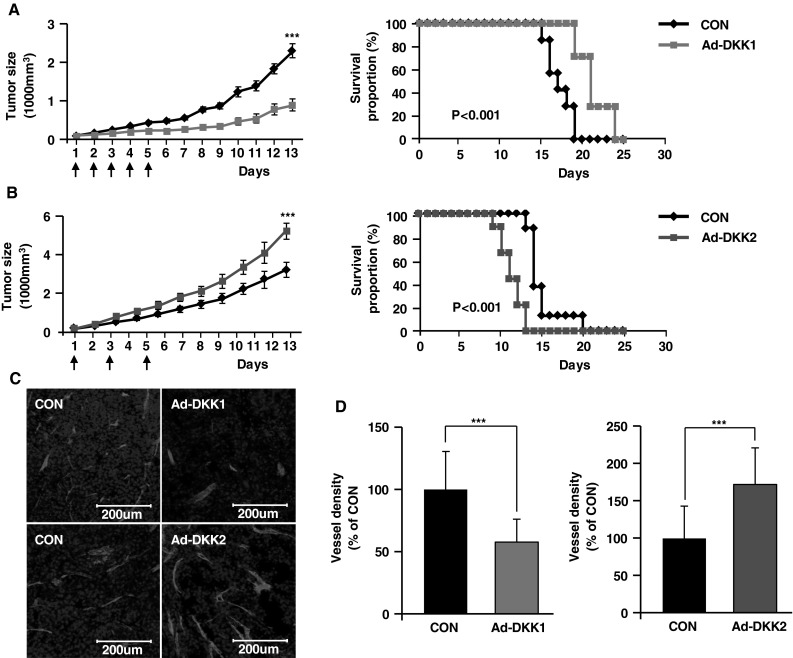
Adenoviral DKK1 and DKK2 expression differentially modulate B16F10 melanoma growth and angiogenesis. B16F10 murine melanoma cells were injected subcutaneously into abdomens of C57BL/6 mice. Mice were injected intratumorally with control adenovirus (CON, n = 8), DKK1-expressing adenovirus (Ad-DKK1, n = 8), or DKK2-expressing adenovirus (Ad-DKK2, n = 8) on the indicated days (*left panels*, *vertical arrows*). Tumor growth was monitored over time, and data are presented as mean ± SE. The percentage of surviving mice was determined by monitoring the tumor growth-related events (tumor size >3,000 mm^3^) over a period of 25 days (**a, b**, *right panels*). 1,000 mm^3^ adenovirus-infected tumors were stained with anti-CD31 antibody, an EC marker. By confocal imaging of tumor sections, vessel density was determined (**c**) and quantified (**d**). Nuclei were stained with DAPI. Data are mean ± SD (***p < 0.001)

To study the effect of DKK1 and DKK2 on tumor angiogenesis and proliferation, we injected either adenoviral-mDKK1 (Ad-DKK1) or -mDKK2 (Ad-DKK2) constructs into B16F10 melanomas that had been established in syngeneic C57BL/6 mice. Intratumoral Ad-DKK1 injection into standard-sized tumors (approximately 100 mm^3^) significantly retarded tumor growth compared to Ad-Mock control construct-infected tumors [874 ± 162 vs. 2,277 ± 186 mm^3^, over 13 days of growth (*p* < 0.001)] (Fig. 1a, left panel). Consequently, Ad-DKK1 administration significantly prolonged tumor-bearing mouse survival (*p* < 0.001) (Fig. 1a, right panel). Conversely, Ad-DKK2 transfection accelerated tumor growth [5,194 ± 422 vs. 3,170 ± 397 mm^3^ (*p* < 0.001)] and shortened survival time in these mice (*p* < 0.001) (Fig. 1b). In a previous study, DKK1 inhibited and DKK2 enhanced angiogenesis both in vitro and in vivo [8]. We hypothesized that DKK1 and DKK2 effects on tumor growth may be due to altered tumor angiogenesis. We analyzed tumor vessel density of Ad-DKK1- and Ad-DKK2-transfected tumors, in comparison with Ad-Mock transfectants. Angiogenesis is a key step for tumor growth, and might be influenced by tumor size. To reduce potential tumor size-associated effects on tumor angiogenesis, tumors were established at approximately 1,000 mm^3^ volumes, and microvascular structures were identified and quantified using EC-specific CD31 immunostaining. Ad-DKK1 infection significantly reduced tumor vessel density (58 % of Ad-Mock controls; Fig. 1c, d). Unlike Ad-DKK1, Ad-DKK2 infection increased B16F10 melanoma vessel density to levels 171 % of Ad-Mock controls (Fig. 1c, d).

### EC-specific over-expression of DKK1 and DKK2 in mice alters in vivo B16F10 melanoma tumor growth and angiogenesis

**Fig. 2:**
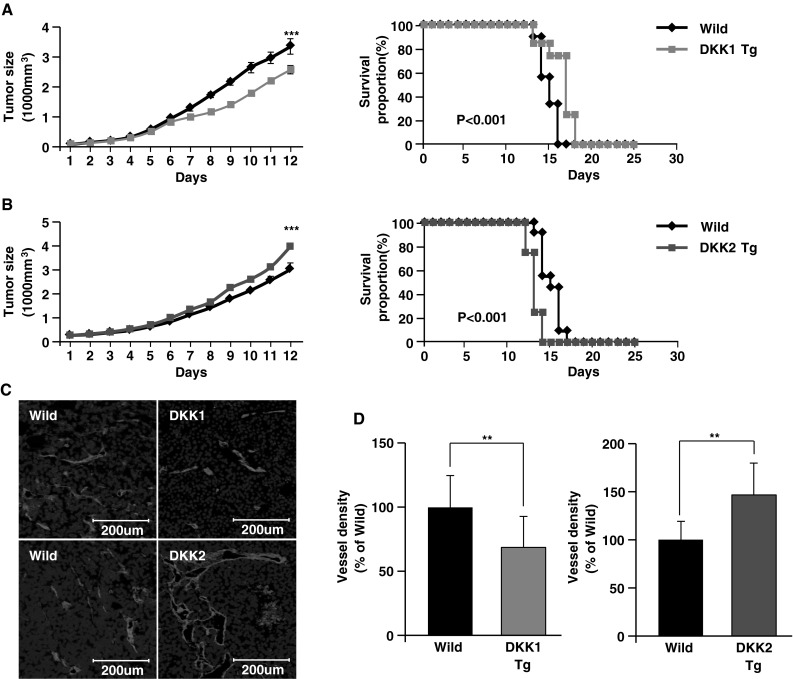
Endothelial-specific DKK1 or DKK2 expression in mouse tumors showed consistent results with the virus model. **a**–**b** B16F10 murine melanoma cells were injected subcutaneously into the abdominal space of transgenic mice that endothelial-specifically expressed DKK1 (DKK1 Tg, n = 10) or DKK2 (DKK2 Tg, n = 11), or their wild-type littermates (n = 10 and n = 11, respectively). Tumor growth was monitored over time, and data are presented as mean ± SE (*left panels*). The percentage of surviving mice was determined by monitoring the tumor growth-related events (tumor size >3,000 mm^3^) over a period of 25 days (*right panels*). Melanoma tumors (1,000 mm^3^) were prepared, excised, and stained with anti-CD31 antibody, an EC marker. Confocal imaging revealed (**c**) and quantified (**d**) CD31^+^ tumor vessels in virus-infected tumor sections. Nuclei were stained with DAPI. Data are mean ± SD (**p < 0.01; ***p < 0.001)

To confirm the effect of DKK1 and DKK2 on tumor angiogenesis, we used DKK1 Tg and DKK2 Tg mice that we generated for our previous study [8]. We injected B16F10 melanoma cells into DKK1 Tg and DKK2 Tg mice, and their wild-type littermates, and evaluated tumor growth and angiogenesis. Tumor size was significantly reduced in DKK1 Tg mice versus wild-type controls (2,590 ± 463 vs. 3,368 ± 856 mm^3^, respectively; Fig. 2a, left panel; *p* < 0.001), which correlated to increased survival in DKK1 Tg mice (Fig. 2a, right panel; *p* < 0.001). On the contrary, melanoma growth was accelerated in DKK2 Tg mice versus wild-type controls (3,844 ± 252 vs. 2,874 ± 140 mm^3^, respectively; *p* < 0.001) and mouse survival was significantly reduced in DKK2 Tg mice (Fig. 2b, right panel; *p* < 0.001). Tumor angiogenesis, as measured by microvascular density, was decreased in DKK1 Tg mice to levels 68 % of those seen in control animals, whereas vessel density in DKK2 Tg mice was increased to levels 127 % of those seen in wild-type animals (Fig. 2c, d).

### DKK1 or DKK2 expression does not alter cell proliferation or survival of B16F10 melanoma cells

**Fig. 3:**
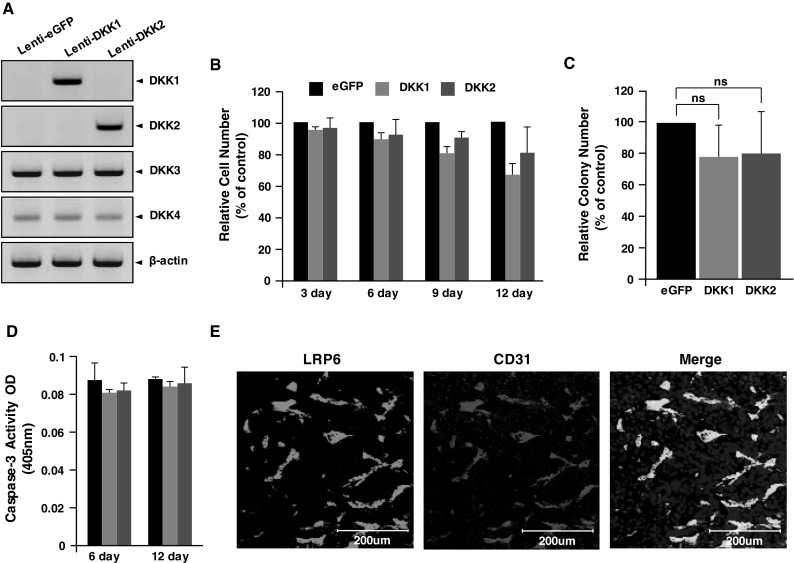
DKK1 or DKK2 expression does not alter tumor cell proliferation, colony formation, or apoptosis. **a** B16F10 tumors were stably transfected with GFP-, DKK1-, or DKK2-expressing lentivirus. Protein levels of DKKs (1–4) were detected in transfectant lysates by western blotting. β-actin was used as a protein loading control. **b** Cell numbers were determined in relation to GFP-expressing cells (set at 100 %) at the indicated times. **c** Colony-forming efficiency of the stable transfectants at 3 weeks after seeding. Data show the number of soft agar colonies relative to GFP-expressing cells (set at 100 %). **d** Stable transfectants were cultured for 6 or 12 days, and caspase-3 activity was measured as an indicator of apoptosis. **e** B16F10 melanomas generated in wild-type mice were frozen-sectioned and immunostained with anti-LRP6 and anti-CD31 antibodies. Data are mean ± SD of triplicate experiments (*ns* not significant; *p* > 0.05)

Altered tumor cell proliferation or survival might affect tumor vessel density. To assess this possibility, we evaluated DKK1 and DKK2 effects on B16F10 melanoma cellular function. DKK1 and DKK2 were undetectable in B16F10 melanoma cells, whereas DKK3 and DKK4 were expressed (Fig. 3a). We constructed stable DKK1 and DKK2 B16F10 melanoma cell lines by transfection with lenti-viral vectors (Lenti-DKK1 and Lenti-DKK2). We generated a stable green fluorescent protein (GFP) transfectant line to use as a control. DKK1 and DKK2 expression in respectively transfected cell lines was confirmed by Western blotting (Fig. 3a). Neither DKK1 nor DKK2 stable melanoma cell line proliferation was significantly different compared to the Lenti-GFP cell line (Fig. 3b). Colony formation after 3 weeks in culture was not altered by DKK1 or DKK2 expression (Fig. 3c). Apoptosis, as indicated by caspase-3 activation, was also not altered by DKK1 or DKK2 expression through 12 days in culture (Fig. 3d). DKK family members bind to LRP5/6 and antagonize canonical Wnt signaling by competitive inhibition [26]. Moreover, DKK2 activates CDC42, which enhances angiogenesis requiring LRP6 expression [8]. Costaining of CD31 and LRP6 in B16F10 melanoma tumors showed vasculature-specific LRP6 expression (Fig. 3e).

Consistently, melanoma blood vessels in DKK1 Tg mouse tumor sections stained less for beta-catenin (37 % reduction) compared with that in control mice (Fig. S1a, b). Unlike DKK1, beta-catenin staining in tumor blood vessels of DKK2 Tg mice was significantly increased compared to wild-type (Fig. S1a, c). There were no differences in beta-catenin expression in other cell types of either DKK transgenic mouse tumor tissues (Fig. S1a, b). Collectively, these results suggest that the effects of DKK1 and DKK2 on B16F10 melanoma growth are mainly exerted through regulating angiogenesis with no direct action on tumor cell proliferation.

### DKK1 and DKK2 differentially modulate tumor vessel functionality and hypoxia

**Fig. 4:**
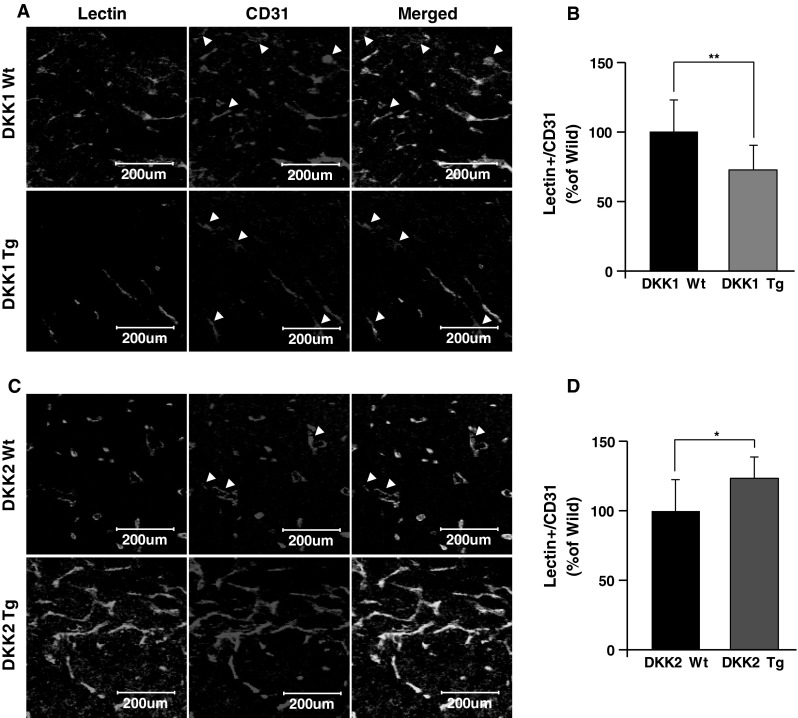
DKK1 inhibits and DKK2 enhances vessel functionality demonstrated by lectin perfusion. DKK1 Tg (n = 6) and DKK2 Tg (n = 6) mice and their wild-type littermates (n = 6 for each group) were subcutaneously injected with B16F10 melanoma cells into the abdomen. Mice bearing 1,000 mm^3^ tumors were injected i.v. with biotinylated lectin. Tumor sections were stained with Alexa488 conjugated to streptavidin and anti-CD31 antibody. Confocal imaging of stained tumor sections revealed lectin-perfused regions (**a**, **c**) and their quantification (**b**, **d**) Data are mean ± SD (*p < 0.05; **p < 0.01)

Tumor vessel functionality is a recently-emerging issue in tumor studies [14–18]. We studied DKK1 and DKK2 effects on tumor vessel functionality by injecting lectin into DKK Tg mice bearing B16F10 tumors and quantifying vessel staining, a surrogate of vascular perfusion, in subsequently harvested tumors. Tumors in DKK1 Tg mice showed a statistically significant decrease in perfused vessel ratio (lectin^+^ vessels/CD31^+^ vessels), to levels 72 % of those seen in control animals (Fig. 4a, b). Conversely, tumor vessel perfusion was increased in DKK2 Tg mouse tumors, to levels 123 % of wild-type littermates (Fig. 4c, d).

**Fig. 5:**
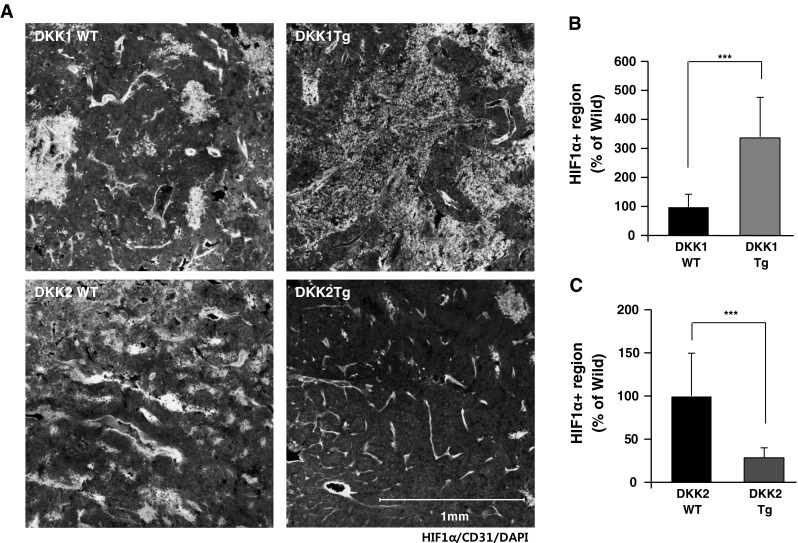
Tumor hypoxia is increased in DKK1 Tg mice and decreased in DKK2 Tg mice compared to wild-type littermates. B16F10 tumor sections of DKK1 Tg, DKK2 Tg, and wild-type littermates were stained with anti-hypoxia-inducing factor-1α (HIF1α) antibody and anti-CD31 antibody. Hypoxic regions were presented (**a**) and quantified as the ratio of HIF1α^+^ tumor area to total tumor area (**b**, **c**)

Inefficient blood perfusion of tumor vessels gives rise to hypoxia, which promotes tumor invasion and metastasis. To examine the effects of DKKs on tumor hypoxia, we stained tumor sections with hypoxia-inducible factor-1α (HIF-1a), an intrinsic marker of hypoxia. DKK1 Tg tumors showed significantly increased hypoxic regions compared to wild-type tumors (207 % of wild-type; Fig. 5a, b). On the contrary, the total hypoxic region was significantly reduced in DKK2 Tg-expressing tumors, to values approximately 25 % of those seen in wild-type tumors. In conclusion, DKK1 and DKK2 regulate tumor vessel functionality, resulting in changes of tumor hypoxia status.

### DKK1 and DKK2 regulate perivascular coverage of tumor vasculature

**Fig. 6:**
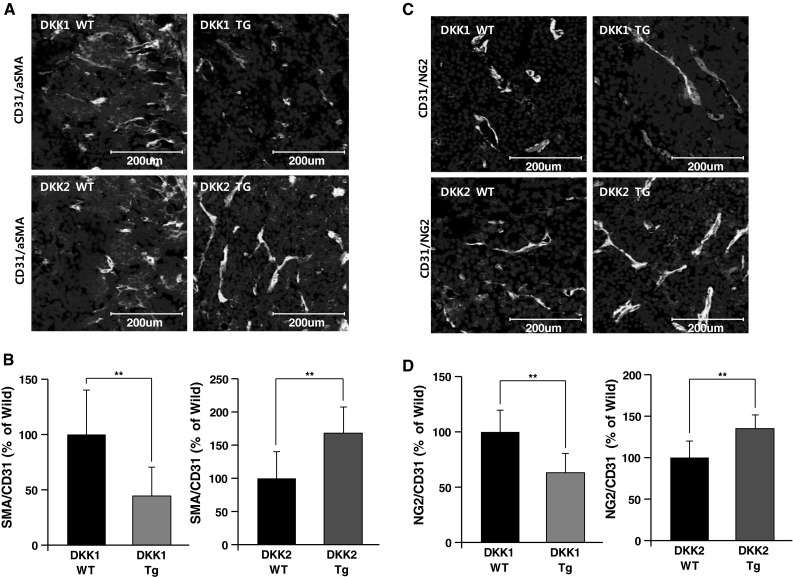
Perivascular cell coverage of tumor vessels is inhibited by DKK1 and enhanced by DKK2. Sections of B16F10 melanoma tumors generated in DKK1 Tg, DKK2 Tg, and wild-type mice were stained with anti-CD31 and anti-smooth muscle actin (SMA) antibodies. Nuclei were stained with DAPI. SMC coverage is presented (**a**) and quantified as the ratio of SMA^+^ area to CD31^+^area (**b**). Sections of tumors generated in DKK1 Tg, DKK2 Tg, and wild-type mice were stained with anti-CD31 and anti-NG2 (pericyte-specific) antibodies. Pericyte coverage is presented (**c**) and quantified as the ratio of NG2^+^ area to CD31^+^ area (**d**). Nuclei were stained with DAPI. Data are mean ± SD (*p < 0.05; **p < 0.01)

Perivascular cell coverage of vessels (i.e., pericytes and SMCs) is critical for stable and mature vascular structure [27]. Impaired perivascular cell coverage causes vascular malfunctions such as leakage and hyperplasia [28]. It is well known that tumor vessels are less-covered compare to normal vessels, and increasing evidence suggests that vessel coverage affects tumor vessel functionality [29]. We hypothesized that altered vascular functionality in B16F10 tumors generated in DKK1 Tg and DKK2 Tg mice was due to changes in perivascular cell coverage. We stained tumor sections with antibodies against smooth muscle actin, NG2, and CD31, which are respective markers for SMCs, pericytes, and ECs. As shown in left panels of Fig. 6a, b, nearly half of tumor vessels in wild-type mice are covered with pericytes or SMCs. Vessels in tumors generated in DKK1 Tg mice had less coverage of pericytes (63 % of wild-type levels) and SMCs (44 % of wild-type; Fig. 6a, b). Conversely, DKK2 Tg mice tumor vessels had more pericyte coverage (134 % of wild-type levels) and SMCs (167 % of wild-type levels; Fig. 6c, d). These results suggest that DKK1 and DKK2 affect tumor vascular functionality in part by altering perivascular coverage.

### DKK1 and DKK2 modulate angiogenesis in an oxygen-induced retinopathy model

**Fig. 7:**
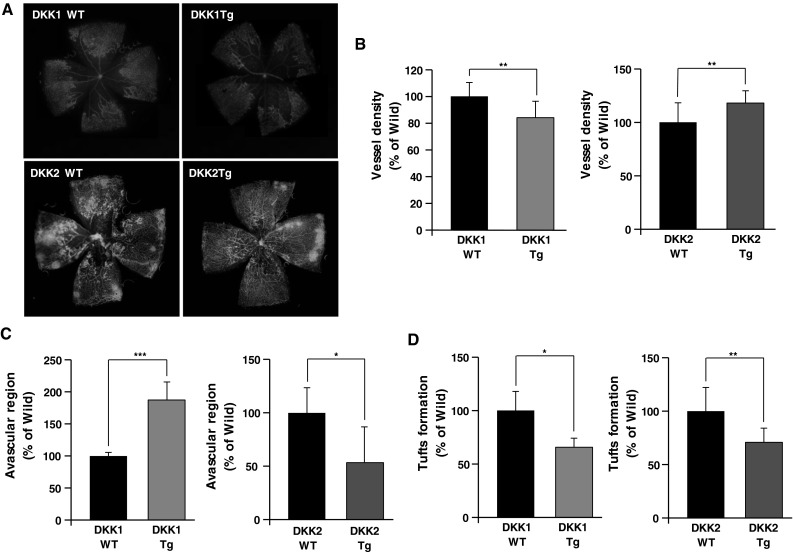
Vessel abnormality caused by oxygen-induced retinopathy (OIR) is decreased in both DKK1 Tg mice and DKK2 Tg mice, compared to wild-type littermates. **a** DKK1 Tg (n = 4), DKK2 Tg (n = 4), and their wild-type littermates (n = 4 in each comparison) were exposed to a 75 % O_2_ environment for 5 days, followed by return to normoxia (i.e., relative hypoxia) for another 5 days. Retinas were assessed after animal sacrifice and enucleation. Fluorescence-conjugated isolectin B4 staining of OIR retina vessels is presented. Vessel density (**b**), avascular region (**c**), and tuft formation (**d**) were quantified. Data are mean ± SD (*p < 0.05, **p < 0.01)

To further investigate potential DKK1 and DKK2 involvement in pathological angiogenesis, we applied an oxygen-induced retinopathy (OIR) model to DKK1 Tg and DKK2 Tg mice. DKK1 treatment is known to decrease retinal tuft formation in a rat OIR model by inhibiting Wnt canonical signaling [30]. In our OIR model, DKK1 Tg mice displayed decreased retinal vessel density (84 % of wild-type levels) and increased avascular area (187 % of wild-type), in agreement with earlier observations in rats (Fig. 7a–c). Tuft formation of DKK1 Tg in the OIR model was reduced to levels 70 % of those seen in wild-type littermates (Fig. 7d). Conversely, DKK2 Tg mice showed significantly increased retinal vessel density (117 % of wild-type levels) in the OIR model (Fig. 7a, b), and decreased avascular regions (to 54 % of wild-type levels; Fig. 7a, c). Interestingly, DKK2 transgenic mice displayed reduced tuft formation (66 % of wild-type levels) in the OIR model as DKK1 Tg mice (Fig. 7a, d). Retinal hemorrhage is the prominent symptom of the OIR model [31]. Both DKK1 Tg and DKK2 Tg mice OIR retinas displayed a minimal incidence of hemorrhage (4 and 6 % of wild-type levels, respectively; Fig. S2).

## Discussion

This study examined the effects of DKK1 and DKK2, known Wnt antagonists, on B16F10 melanoma tumor proliferation and angiogenesis by using viral-mediated tumor transfectants and transgenic mice. Neither DKK1 nor DKK2 affect B16F10 melanoma cell proliferation and survival. However, DKK1 inhibits tumor growth by decreasing tumor angiogenesis and vascular perfusion. Interestingly, DKK2, a homolog of DKK1, increased tumor neovessel formation and perfusion, consequently accelerating tumor growth. These findings are consistent with our previous study that demonstrated that DKK2 positively regulates, whereas DKK1 inhibits, angiogenesis both in vitro and in vivo [8].

The role of DKK1 in tumor growth regulation has been suggested in several tumor models. DKK1 is epigenetically silenced in several cancers such as human melanoma and renal cell carcinoma [10]. Ectopic DKK1 expression suppressed tumor growth by inducing apoptosis and inhibiting proliferation in both renal cell carcinoma and MDA-MB-435 melanoma [12]. Although most of these studies demonstrated an inhibitory role of DKK1 on tumor growth, the effect of DKK1 on vessel invasion into tumors, which is essential to tumor growth and metastasis, has not been intensively studied. Our findings suggest that DKK1 decreases B16F10 tumor growth through regulating the tumor vasculature. Conversely, one study showed that DKK1 enhances glioma-tumor angiogenesis by negatively regulating delta-like ligand-4 [32]. The discrepancy between glioma tumor results and our B16F10 tumor data may be explained by the complexity of Wnt signaling components and different DKK1 delivery methods. Some papers suggest that blocking Wnt signaling induces angiogenesis [33, 34]. Other papers demonstrated that Wnts, including Wnt3a and Wnt5a, stimulate proliferation and enhance tube formation of cultured human ECs [5, 35–37]. Moreover, previous our data showed that DKK1 inhibits angiogenesis in vitro and in vivo. In line with this observation, other Wnt antagonists (e.g., sFRP1 and WIF1) inhibit tumor angiogenesis in hepatocellular carcinoma by preventing both EC proliferation/migration and endothelial precursor cell differentiation [38]. Although additional work is required to clarify the effects of DKK1 on tumor angiogenesis, our results consistently support that Wnt regulates tumor angiogenesis. Interestingly, DKK2 increased tumor angiogenesis, which was correlated with accelerated B16F10 tumor growth. DKK1 and DKK2 have high structural similarity; however, while DKK1 inhibits Wnt signaling, DKK2 activates Wnt canonical signaling in a context-dependent manner in *Xenopus* species [39]. That DKK2, but not DKK1, activates tumor angiogenesis is supported by the observation that DKK2 enhances retinal angiogenesis and induces neovessel formation in several ex vivo assays [8]. Together, these data demonstrate that DKK1 negatively, and DKK2 positively, regulates tumor angiogenesis.

Alteration of tumor cell proliferation affects tumor growth which might affect tumor angiogenesis. Even more, Wnt/beta-catenin signaling is known to regulate proliferation of several tumor cell types [40, 41]. The DKK family is known to inhibit Wnt/beta-catenin signaling by binding to the Wnt co-receptor, LRP6 [26]. However, we found that both DKK1 and DKK2 did not modulate tumor cell proliferation in stably-transfected melanoma cells. In line with this finding, beta-catenin staining of tumors showed that DKK1 and DKK2 altered beta-catenin accumulation in vessels but not in other cells. Vessel-specific LRP6 expression in tumors supports the idea that DKK1 and DKK2 expression does not affect tumor cell proliferation. KREMEN2 is necessary for DKK1-mediated Wnt inhibition [42]. In B16F10 tumor sections, KREMEN2 was expressed in essentially all cell types, including ECs (Fig. S3). Thus, vessel-specific expression of LRP6 might limit any potential proliferative effect by DKK1 and DKK2 in B16F10 tumors. Even more, VEGF-A expression of B16F10 did not altered by DKK1 and DKK2 treatments (Fig. S4). Taken together, our data indicate that DKK1 and DKK2 effects on tumor growth involve DKK-modulated angiogenesis and altered tumor neovascular structure.

Increasing attention is being given to approaches to normalize tumor microvascular structure and perfusion, to prevent tumor metastasis and enhance chemotherapy [43–46]. Our findings show that DKK1 inhibits vessel perfusion and reduces perivascular coverage in B16F10 melanoma tumors. Perivascular coverage by SMCs and pericytes is an important component of functional vasculature [27]. Pericyte loss leads to abnormal EC membrane folding and irregular vascular diameters that reduce vessel perfusion [47]. There is growing evidence that DKK1 negatively regulates pericyte and SMC proliferation. Ren et al. [22] suggested that LRP6 is indispensible for PDGF-BB-induced pericyte proliferation, and DKK1 inhibits this effect in vitro and in vivo. Although that study showed that DKK1 inhibits pericyte detachment in a kidney injury model, the total number of vascular pericytes is reduced by circulating DKK1. In tumor growth, neovessel formation occurs rapidly, unlike in the kidney injury model. Even more, PDGF receptor expression by SMCs and pericytes is dependent on Wnt/beta-catenin signaling, and is more important than PDGF expression for SMC and pericyte recruitment to vessels [48]. Although there are no reports about DKK1 direct effects on SMCs, LRP6 blockade inhibits, and Wnt1 induces, SMC proliferation [49], suggesting that DKK1 negatively regulates SMC proliferation. In line with these reports, Wnt1 enhances tumor vessel coverage in gliomas via increasing endothelial PDGF-B secretion, whereas DKK1 inhibits this effect [32]. These reports suggest that DKK1-inhibited vessel perfusion, which induced perivascular hypoxia in tumors, occurs in part by inhibiting perivascular cell proliferation leading to poor vascular coverage and abnormal vessel structure.

Opposite to DKK1, DKK2 enhances pericyte and SMC coverage of B16F10 tumor vessels. DKK2-induced vessels are more completely covered compared to VEGF-induced vessels in a cornea implant assay, supporting that DKK2 enhances perivascular cell coverage of growing vessels [8]. Nevertheless, the detailed mechanisms of DKK2-regulated perivascular cell coverage of tumor neovessel remain to be elucidated. LRP6 mediates DKK2-induced CDC42 activation, and LRP6 is expressed in both pericytes and SMCs, suggesting that DKK2 has a potential for regulating perivascular cell function [22, 50]. This may underlie the observed DKK2-mediated increases in both vessel perfusion and vessel density, and subsequently reduced hypoxia, in DKK2 Tg mouse bearing tumors.

In our OIR model, DKK1 reduced retinal neovessel density and vascularized area, similar to effects seen in tumors grown in DKK1 transgenic mice. LRP6 expression increases in retinal ECs in the OIR model, and DKK1 treatment reduces retinal tuft formation and vessel density by blocking Wnt-promoted EC proliferation in rat OIR model retinas [51]. We showed that hemorrhage of the OIR retina is reduced in DKK1 Tg mice. This effect might be related to the reduction of tuft formation [52] and inhibitory effect of DKK1 on VEGF and Wnt signaling [8]. An interesting finding was that while retinal vessel density increased in the peripheral vascularized region of OIR DKK2 Tg mice, tuft formation in these animals also decreased. Tufts form during rapid vessel growth by abrupt growth factor induction in avascular regions upon return to normoxia, where vessels had previously regressed during hyperoxia. The Tie2 promoter that we used for the DKK2 overexpression is induced by hyperoxic conditions [53]. Thus, we speculate that suppressed vessel regression because of DKK2 overexpression during hyperoxia may lessen the relative hypoxic condition present upon return to normoxia, resulting in both less tuft formation and a lower incidence of retinal hemorrhage.

In conclusion, our data indicate that DKK1 and DKK2 regulate both tumor angiogenesis and perivascular coverage. Because improved vascular functionality is the aim of tumor vessel normalization approaches, DKK1 and DKK2 effects on angiogenesis and vessel functionality that regulates tumor hypoxia might be purposefully exploited in developing novel cancer treatments.

## Electronic supplementary material

Below is the link to the electronic supplementary material.

Supplementary material 1 (PDF 314 kb)
