# Supplementary material for: Distinct roles of DKK1 and DKK2 in tumor angiogenesis
Source: Angiogenesis. 2013 Oct 4;17(1):221–34. doi: 10.1007/s10456-013-9390-5 (PMC3898121; doi:10.1007/s10456-013-9390-5)

Distinct roles of DKK1 and DKK2 in tumor angiogenesis

♦*Angiogenesis*

[1]Hongryeol Park, [1]Hyei Yoon Jung, [1]Hyun-Jung Choi, [1]Dong Young Kim, [2]Ji-Young Yoo, [2]Chae-Ok Yun, [3]Jeong-Ki Min, [4]Young-Myoung Kim, and [1]Young-Guen Kwon

Young-Guen Kwon, Ph.D.
Department of Biochemistry
College of Life Sciences and Biotechnology
Yonsei University,
Seoul, 120-749,
Republic of Korea.
e-mail: ygkwon@yonsei.ac.kr
phone: 82-2-2123-5697
Fax: 82-2-362-9897

SUPPLEMENTAL FIGURE LEGENDS

Supplementary Figure 1. DKK1 decreased and DKK2 increased beta-catenin accumulation. a-b) Sections of B16F10 melanoma tumors generated in DKK1 Tg, DKK2 Tg, and wild-type mice were stained with anti-beta-catenin and anti-CD31 antibodies. Nuclei were stained with DAPI. Beta-catenin staining is represented **(a)** and quantified as the ratio of beta-catenin^{+}EC/ EC **(b, c)**.

Supplementary Figure 2. Retinal hemorrhage was reduced in DKK1 Tg and DKK2 Tg mice in an oxygen-induced retinopathy model. a-b) DKK1 Tg (n=6), DKK2 Tg (n=6) mice and their wild-type littermates (n=6 in each group) were subjected to hyperoxia in an oxygen-induced retinopathy model. Retinas were observed for hemorrhage **(a)** that was quantified **(b)**.

Supplementary Figure 3. Kremen2 was expressed in overall cells including ECs. B16F10 tumor section of Wild mouse was stained with anti-Kremen2 and anti-CD31. Nuclei were stained with DAPI.

Supplementary Figure 4. DKK1 and DKK2 did not alter VEGF-A expression of B16F10. After 4hr starvation, DKK1 (200ng/ml) or DKK2 (200ng/ml) were treated at B16F10 for the indicated times. Protein levels of VEGF-A were detected in whole cell lysates by western blotting. β-actin was used as a protein loading control.

Supplemental Fig. 1

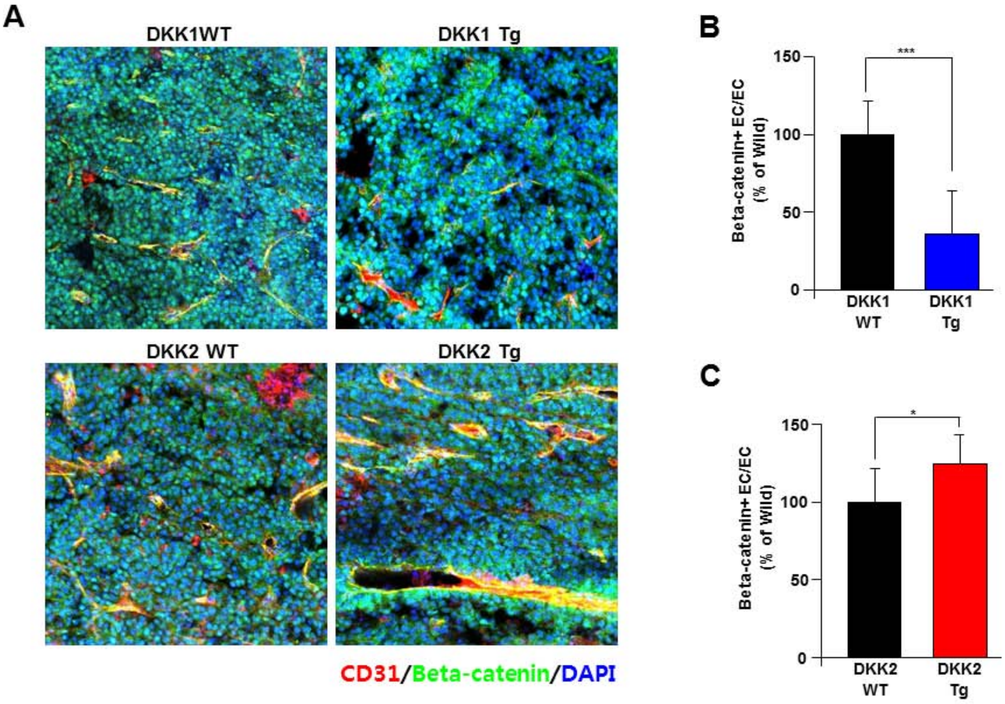

Supplemental Fig. 2

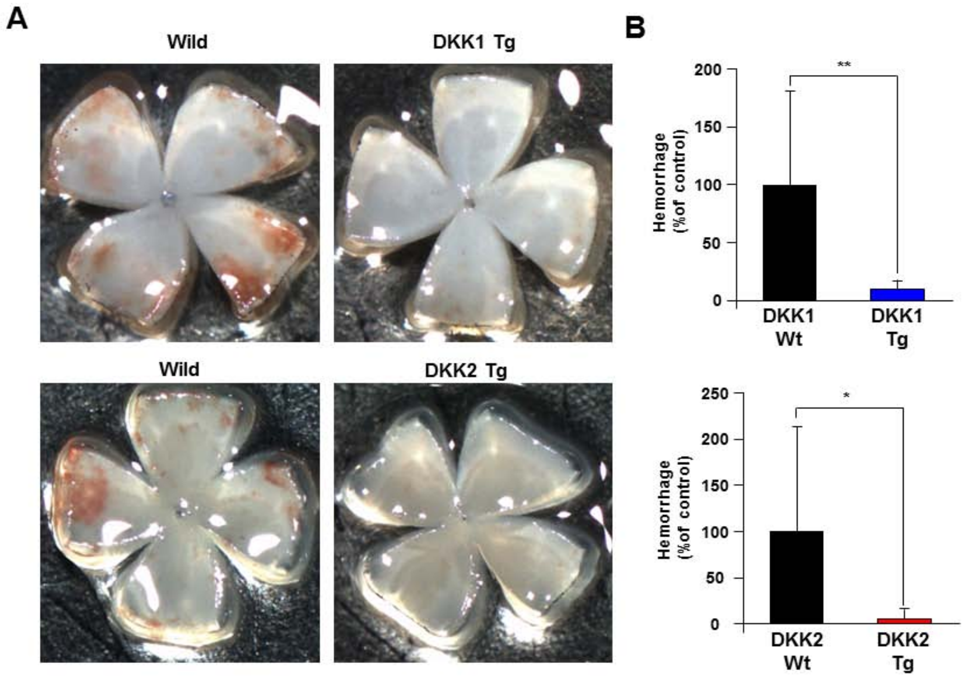

Supplemental Fig. 3

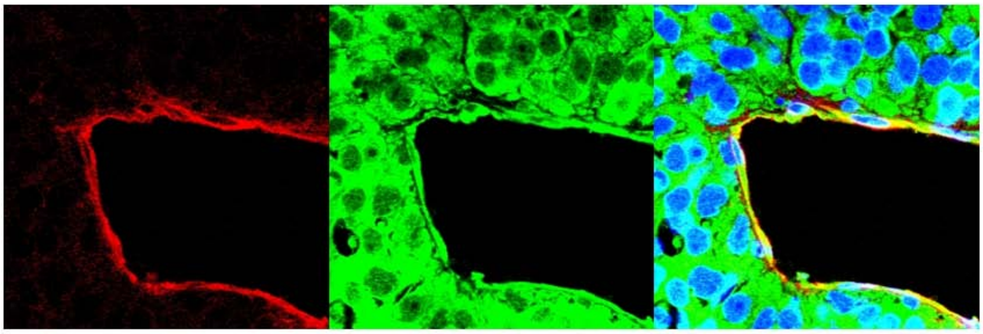

Supplemental Fig. 4

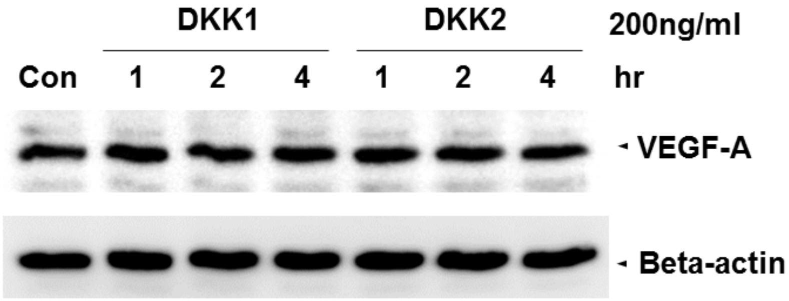

Supplement: Supplementary file 1 — Supplementary material 1 (PDF 314 kb) [file 10456_2013_9390_MOESM1_ESM.pdf]
